# Pangenome and Pan‐Transcriptome Analysis of the *WOX* Gene Family Reveals Evolutionary Conservation and Diversification in Alfalfa

**DOI:** 10.1002/ece3.74048

**Published:** 2026-08-04

**Authors:** Hao Wen, Yiwei Bai, Zhao Guo, Wenwu Qian, Yizhi Huang, Yutao Liang, Guodong Yang, Yunwei Zhang

**Affiliations:** ^1^ College of Grassland Science and Technology China Agricultural University Beijing China

**Keywords:** alfalfa, orthologous gene groups, pan‐genome, pan‐transcriptome, WOX family

## Abstract

The *WUSCHEL‐related homeobox* (*WOX*) gene family encodes plant‐specific transcription factors that play pivotal roles in meristem maintenance, organogenesis, regeneration, and developmental phase transitions. Despite their importance, the pangenome‐scale composition, expansion dynamics, and evolutionary constraints of *WOX* genes in alfalfa ( *Medicago sativa* ) remain poorly understood. Here, we performed an integrated pangenome and pan‐transcriptome analysis of the *WOX* gene family using 24 high‐quality alfalfa genomes. A total of 432 *WOX* genes were identified and clustered into 24 orthologous gene groups (OGGs). Pangenome profiling revealed that the WOX family is highly conserved across alfalfa accessions, with members of the Ancient clade showing greater conservation than those of the Intermediate and WUS clades. Duplication pattern analysis indicated that dispersed duplication was the primary force driving WOX family expansion, whereas whole‐genome duplication (WGD) events predominantly contributed to the long‐term retention of core and softcore members. Pairwise Ka/Ks analysis indicated strong purifying selection on most WOX gene pairs, whereas wild‐germplasm genes and Intermediate‐clade members showed elevated selective pressures. Notably, MsWOX9‐related homologs had exceptionally high Ka/Ks values, suggesting potential functional divergence linked to agronomically relevant traits. Furthermore, pan‐transcriptome analysis revealed that *WOX* genes could be classified into three distinct expression patterns during leaf senescence. Collectively, this study presents the first comprehensive pangenome‐ and pan‐transcriptome‐based characterization of the WOX family in alfalfa, providing new insights into its evolutionary dynamics and expression divergence.

## Graphical abstract

In this study, we conducted the first integrated pangenome and pan‐transcriptome analysis of the WOX gene family in alfalfa using 24 high‐quality accessions and 43 transcriptome datasets. Our results reveal strong evolutionary conservation dominated by core and soft‐core members, clade‐specific diversification driven mainly by dispersed duplication, and distinct developmental expression patterns, highlighting regulatory‐level functional divergence.

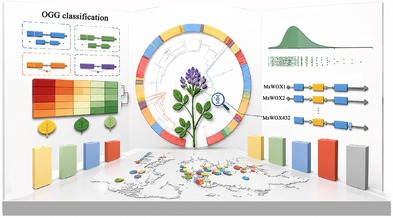

## 1 Introduction

*Medicago sativa* L. (alfalfa) is one of the most important leguminous forage crops worldwide and widely regarded as the “king of forages” for its high protein content, broad environmental adaptability, and capacity for biological nitrogen fixation. With the continuous advancement of alfalfa molecular breeding technologies and the increasing application of genomic‐assisted improvement strategies in alfalfa (Hawkins and Yu 2018), elucidating the roles of key functional gene families in growth, development, and environmental adaptation has become a central objective for dissecting genetic mechanisms and accelerating cultivar improvement.

With the advancement of high‐throughput and long‐read sequencing technologies, pangenome research has become a major frontier in genomics. By integrating genomic data from multiple individuals, pangenomes enable the identification of core and variable genes at the species level, providing a more comprehensive view of intraspecific genetic diversity. In plants, pangenome studies in crops such as maize, rice, and wheat have revealed extensive gene presence–absence variation and structural variation, highlighting the importance of pangenomes for understanding genetic diversity and evolutionary processes (Jiao et al. 2024; Qian et al. 2025; Yang et al. 2025).

The *WUSCHEL‐related homeobox (WOX)* family of genes encodes plant‐specific transcription factors and represents an important branch of the Homeobox superfamily. Based on their phylogenetic relationships, *WOX* genes are classified into three major clades: the modern (WUS) clade, the intermediate clade, and the ancient clade (van der Graaff et al. 2009). WOX transcription factors function as key developmental regulators and participate in a wide range of biological processes, including meristem maintenance, organogenesis, embryonic patterning, callus formation, and responses to abiotic stresses, thereby exerting central roles throughout the plant life cycle (Zhang et al. 2024; Zuo et al. 2002).

As a perennial forage crop, alfalfa has several important traits, including sustained leaf growth, regrowth after cutting, root development, stress adaptation, and forage quality; these processes may be closely associated with WOX‐mediated regulation of meristem activity, organogenesis, and leaf senescence. For instance, *MtWOX9‐1*, a member of the intermediate clade, strongly promotes somatic embryogenesis, and its overexpression can even induce embryogenic competence in non‐embryogenic tissues (Yakovleva et al. 2024). In addition, *WOX1* and *WOX9* coordinately regulate cell proliferation and differentiation, thereby controlling leaf development and playing essential roles in leaf organogenesis (Wang et al. 2022). Moreover, *WOX5* exhibits nodule‐specific expression in *Medicago truncatula* and is involved in nodulation organs formation (Mortier et al. 2010).

In cultivated alfalfa, accumulating evidence further highlights the importance of *WOX* genes in agronomically relevant traits. A genome‐wide association study (GWAS) identified several WOX family members as high‐confidence candidate genes significantly associated with root development and root biomass, implicating their roles in regulating root system architecture and influencing forage yield (Jiang et al. 2025). Furthermore, functional analyses have demonstrated that *MsWOX13* enhances waterlogging tolerance in alfalfa under stress conditions (Subedi et al. 2025).

To date, the *WOX* gene family has been systematically identified in a wide range of plant species, including rice and maize, and these studies have laid an important foundation for elucidating the evolutionary relationships and potential functions of WOX transcription factors (Yin et al. 2023; Zhang et al. 2010). In several species, WOX research has extended beyond single reference genomes to multi‐accession comparative analyses or pangenome‐scale investigations. For instance, a comprehensive phylogenetic analysis of 330 *WOX* genes from 29 plant species, including *spanning algae*, bryophytes, ferns, gymnosperms, and angiosperms, revealed that *WUS* genes had already emerged in ferns, highlighting the ancient evolutionary origin of the *WOX* gene family (Liu et al. 2025). At cultivar levels, comparative analyses have also attracted increasing attention. A recent study across 12 cucumber cultivars revealed substantial variation in *CsWOX* genes in terms of protein length, gene structure, and conserved domains, reflecting divergent evolutionary trajectories during adaptation to distinct cultivation environments.

In contrast, in alfalfa, studies of the *WOX* gene family have largely been limited to reference genomes or a limited number of accessions, and systematic analyses within a pangenome framework are still lacking (Yin et al. 2023). Given the high heterozygosity, polyploid genetic background, and pronounced inter‐cultivar genetic diversity of alfalfa, the *WOX* gene family may exhibit unique patterns of gene composition, evolutionary conservation, and variability that differ from those observed in model plant species. Therefore, a comprehensive pangenome‐scale identification of *WOX* genes in alfalfa, together with the delineation of orthologous gene groups (OGGs) and the characterization of core and variable gene components, is essential for gaining deeper insights into the evolutionary dynamics and functional potential of the WOX family in perennial forage crops.

Recently, a comprehensive alfalfa ( *Medicago sativa* ) pangenome dataset comprising 24 accessions was released, including cultivated varieties, landraces, and wild relatives, which provides an important foundation for systematically dissecting gene family composition and genetic variation in alfalfa (He et al. 2025). Building on this resource, the present study focuses on the *WOX* gene family and performs a systematic identification and characterization of *WOX* genes within a pangenome framework. Specifically, we comparatively analyzed the structural features, duplication patterns, and evolutionary dynamics of *WOX* genes across different OGGs and evolutionary clades and further integrated pangenome‐based transcriptomic data to investigate the expression patterns of *WOX* genes during leaf growth and development. Collectively, this study establishes a comprehensive genomic resource for understanding the evolutionary diversification and regulatory differentiation of WOX transcription factors in alfalfa and provides a theoretical foundation for their potential application in alfalfa molecular breeding programs.

## 2 Materials and Methods

### 2.1 Identification of *WOX* Gene Family Members in the Alfalfa Pangenome

The genome assemblies of 24 alfalfa ( *Medicago sativa* ) accessions were obtained from the publicly available dataset hosted on Figshare (https://figshare.com/articles/dataset/Alfalfa/28426967) (Table S1). Because no coding sequences (CDSs) annotations were available in the GFF file, CDS were predicted from transcript sequences using TransDecoder v5.7.1. To identify candidate WOX family members, hidden Markov model (HMM) searches were performed using HMMsearch (v3.3.2) to screen amino acids sequences containing the conserved homeodomain (PF00046) (Potter et al. 2018), In parallel, BLAST+ (2.17.0) searches were conducted by aligning protein sequences from the 24 alfalfa genomes against *Arabidopsis* WOX protein sequences downloaded from PlantTFDB (https://planttfdb.gao‐lab.org/index.php), with an *E*‐value threshold of ≤ 1 × 10^−5^. All candidate proteins obtained from these analyses were further validated by conserved domain identification using the NCBI Conserved Domain Search (CD‐search) tool (https://www.ncbi.nlm.nih.gov/Structure/bwrpsb/bwrpsb.cgi?cdsid=QM3‐qcdsearch‐2A04DB96EC3238C1&tdata=qopts).

### 2.2 Classification of the *WOX* Gene Family in Alfalfa

#### 2.2.1 Classification of Core, Non‐Core Genes, and PCA Analysis

OGG analysis was performed for the identified *WOX* gene family members using OrthoFinder (v2.5.4) (Emms and Kelly 2019). Principal component analysis (PCA) was performed using ImageGP based on the WOX orthogroup gene count matrix generated by OrthoFinder to assess differences in *WOX* gene composition among the 24 alfalfa accessions.

#### 2.2.2 Multiple Sequence Alignment and Phylogenetic Tree Construction

For each OGG, the longest protein sequence was selected as the representative for phylogenetic analysis. Protein sequences were aligned using MUSCLE (v3.8.31) to examine sequence variation within conserved domains (Edgar 2004), The resulting alignments were subsequently trimmed using trimAl (v1.5.1) to remove poorly aligned regions (Capella‐Gutiérrez et al. 2009). Maximum‐likelihood phylogenetic trees were then inferred using IQ‐TREE (v2.4.0). To facilitate clade assignment and evolutionary interpretation, WOX protein sequences from *Arabidopsis thaliana* were included as references in the phylogenetic analysis (Minh et al. 2020). In addition, WOX family members from *Arabidopsis thaliana* , *Oryza sativa* , *Picea abies* , *Physcomitrium patens*, fern species, and *Selaginella moellendorffii*, together with all WOX members identified from the 24 alfalfa genotypes, were used to construct a phylogenetic tree and perform comparative evolutionary analysis of the alfalfa *WOX* gene family.

#### 2.2.3 Motif Analysis and Cis‐Acting Element Analysis of the *WOX* Gene Family

For motif analysis, the gene with the longest CDS was selected as the representative from each OGG. Conserved motifs of the representative WOX proteins were identified using the MEME Suite, with the ZOOPS model selected and the maximum number of motifs set to 10 (Bailey et al. 2009). To analyze cis‐regulatory elements, the 2000 bp upstream sequences from the translation start site of all *WOX* genes were extracted using TBtools (v2.400) and subsequently submitted to the PlantCARE database for cis‐acting element prediction (Chen et al. 2023).

#### 2.2.4 Gene Duplication and Expansion Analysis

To assess sequence similarity among members of the *WOX* gene family, an all‐against‐all BLAST analysis was performed using the *WOX* gene datasets derived from 24 accessions. To ensure the reliability of homology inference, BLAST results were stringently filtered using the following criteria: an *E*‐value ≤ 1 × 10^−5^, sequence similarity ≥ 50%, and retention of only the top 10 hits for each query sequence. Based on the filtered BLAST results, syntenic blocks and collinear gene pairs within the alfalfa pangenome were systematically identified using MCScanX (v1.1).

To further characterize the expansion and duplication patterns of the *WOX* gene family, the duplicate_gene_classifier module in MCScanX (v1.1) was used to classify duplication types (Wang et al. 2012).

### 2.3 Selection Pressure Analysis

For each OGG, one‐to‐one homologous gene pairs were constructed to ensure unbiased estimation of selection pressure. Specifically, based on the phylogenetic tree inferred from protein sequences, the closest homolog for each gene was identified according to genetic distance, and each gene was used only once to form a unique homologous gene pair. Subsequently, selection pressure acting on these homologous gene pairs was evaluated using the nonsynonymous/synonymous substitution rate ratio (Ka/Ks) Calculator (v3.0). The nonsynonymous substitution rate (Ka), synonymous substitution rate (Ks), and their ratio (Ka/Ks) were calculated for each gene pair. Furthermore, the Ka/Ks values of *WOX* gene family members were summarized and averaged separately for cultivated varieties, landraces, and wild alfalfa accessions, enabling comparative assessment of selective constraints across different domestication types (Zhang 2022).

### 2.4 Pangenome‐Based Transcriptomic Expression Analysis

To investigate the regulatory potential of *WOX* genes in alfalfa leaf development, a total of 43 leaf transcriptome datasets representing different developmental stages were collected (Table S2) (Feyissa et al. 2020; He et al. 2025; Ma et al. 2021; Yuan et al. 2020). Representative gene sequences from the 24 OGGs were used as references for transcript quantification. Expression levels were quantified using Kallisto, and the resulting expression matrix was row‐wise scaled to enable comparative analysis of relative expression patterns of OGG representative genes across young, mature, and senescent leaves (Bray et al. 2016).

### 2.5 Expression Pattern Analysis

To analyze the tissue‐specific expression patterns of *WOX* gene family members, samples were collected from young leaves, mature leaves, and old leaves of the same alfalfa plant. Total RNA was extracted using an RNA extraction kit according to the manufacturer's instructions (Huayueyang, China). First‐strand cDNA was synthesized using a reverse transcription kit (Takara, Japan). Quantitative real‐time PCR was performed using TB Green reagent (Takara, Japan) on a real‐time PCR detection system. Three biological replicates were included for expression analysis. All primer sequences used in this assay are listed in Table S3.

## 3 Results

### 3.1 Identification of the *WOX* Gene Family in the Alfalfa Pangenome

**FIGURE 1:**
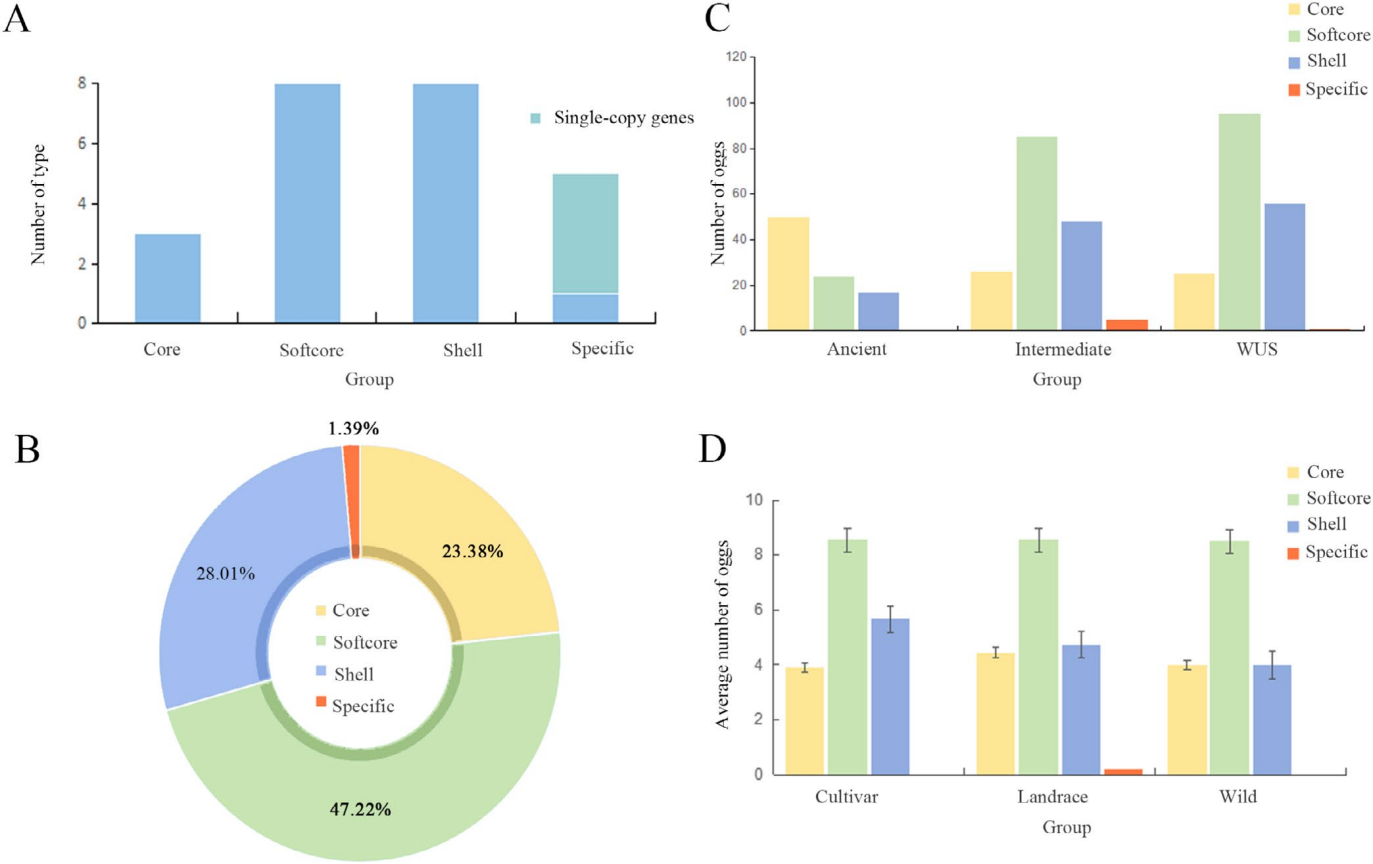
Number and Classification of *WOX* Genes in Alfalfa. (A) Number of OGGs of *WOX* in each component; (B) Percentage of each OGG gene; (C) The number of OGG genes in each clade; (D) Number of OGGs in different types.

A systematic survey of the *WOX* gene family was performed across the genomes of 24 alfalfa accessions, resulting in the identification of a total of 432 *WOX* genes. The number of *WOX* genes per accession ranged from 15 to 23. When grouped by germplasm type, cultivars and landraces exhibited nearly identical patterns. Across materials from different geographical origins, the number of *WOX* genes varied only slightly, with the average values in most regions concentrated between 16 and 19 (Figure S1). Based on their presence–absence patterns across the alfalfa pangenome, *WOX* genes were classified into four categories: core genes, present in all genomes; softcore genes, present in at least 90% of the genomes; shell genes, present in 10%–90% of the genomes; and specific genes, present in fewer than 10% of the genomes. Based on orthology analysis, all identified genes were classified into 24 OGGs (Table S4). Among these OGGs, three were classified as core OGGs, eight as soft‐core OGGs, eight as shell OGGs, and five as specific OGGs. Notably, the specific category included four single‐copy genes (Figure 1A).

The PCA results showed that cultivars and landraces largely overlapped in the PCA space, with no clear separation among different material types, indicating that the WOX OGG composition was generally stable across different alfalfa accessions. A few accessions deviated from the central cluster, suggesting accession‐specific differences in WOX copy number (Figure S2).

Across the pangenome, 70.6% of *WOX* genes belonged to the core and soft‐core categories. The soft‐core category accounted for the largest number of genes, with 204 genes (47.22% of the total), whereas the specific category was the smallest, containing only five genes (Figure 1B).

At the subfamily level, *WOX* genes exhibited pronounced differences in OGG composition. The ancient subfamily was predominantly composed of core OGGs, with a total of 50 core genes. In contrast, the intermediate subfamily was enriched in soft‐core OGGs, which included 85 genes and markedly exceeded the numbers of shell (48 genes) and core (26 genes) OGGs; only five specific genes were identified in this subfamily. The WUS subfamily was mainly composed of soft‐core and shell OGGs, containing 95 and 56 genes, respectively (Figure 1C).

Comparative analysis among different alfalfa material types revealed relatively small differences in the average numbers of *WOX* genes among cultivated varieties, landraces, and wild accessions. Specifically, cultivated varieties contained an average of 3.889 core genes and exhibited a higher number of shell genes than landraces and wild accessions. In contrast, the numbers of soft‐core genes were consistent across all three groups, remaining around 8.5 per accession. Notably, specific genes were detected exclusively in wild accessions and were absent from both cultivated varieties and landraces (Figure 1D).

**FIGURE 2:**
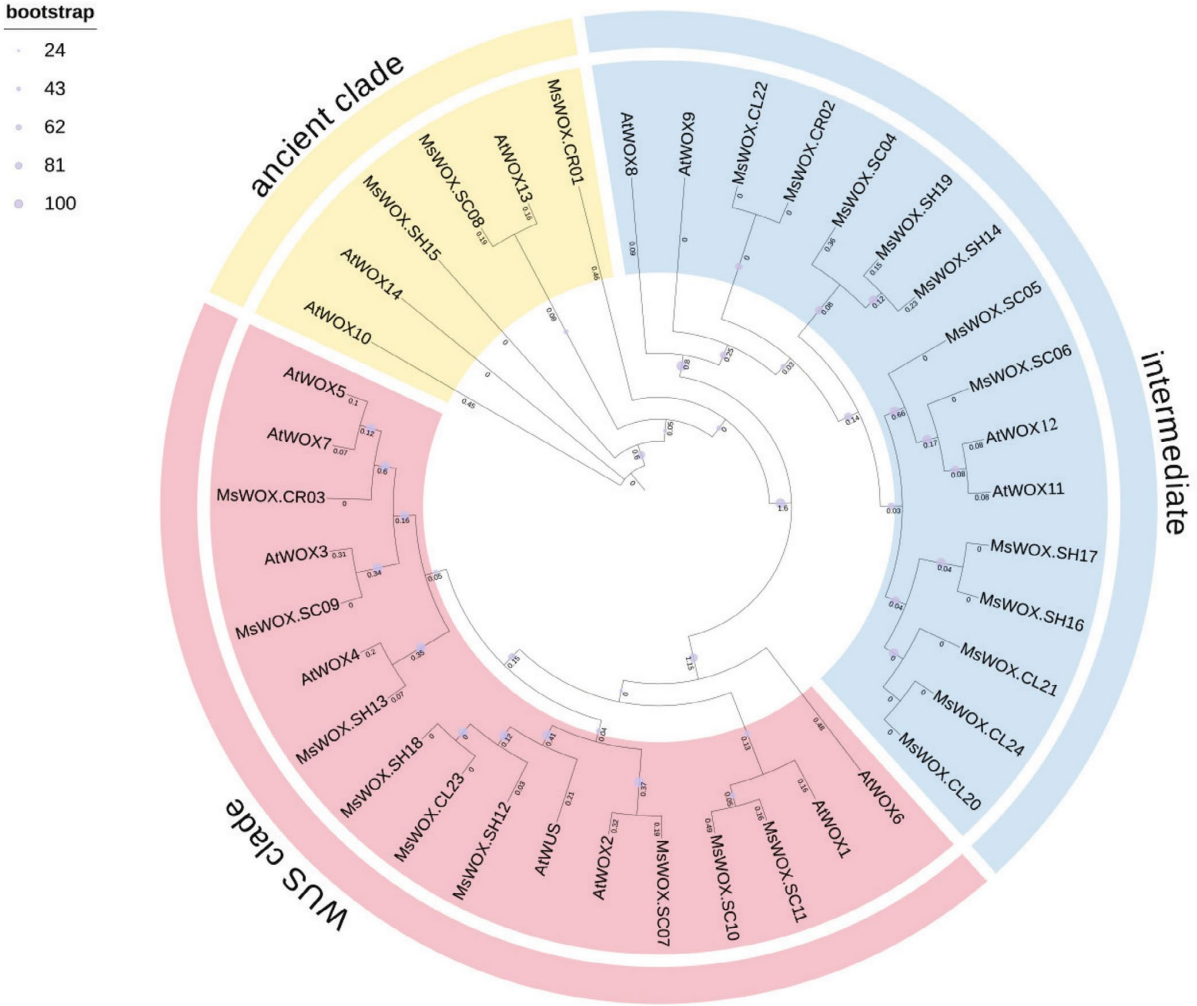
Phylogenetic tree of representative genes from OGGs in the *WOX* gene family. CL, representative genes from specific OGGs; CR, representative genes from core OGGs; SC, representative genes from soft‐core OGGs; SH, representative genes from shell OGGs.

To ensure representative phylogenetic inference, the longest protein sequence from each OGG was selected to construct a phylogenetic tree (Table S5). Based on this analysis, alfalfa *WOX* genes were clearly classified into three major clades: the ancient clade, the intermediate clade, and the WUS clade (Figure 2). The ancient clade contained a small number of members, comprising only three representative genes derived from core, soft‐core, and shell OGGs, and exhibited a compact branching pattern. The intermediate clade accounted for the largest proportion of WOX members and further diverged into multiple sub‐branches. In contrast, the WUS clade formed a distinct and tightly‐knit lineage, clearly separated from the other two clades (Figure 2). Notably, core OGGs were divided into three groups, with one core OGG representative present in each WOX clade. Consistently, the expanded phylogenetic tree including all alfalfa WOX proteins and WOX homologs from representative plant species recovered the same three‐clade topology, further supporting the reliability of the OGG‐based classification (Figure S3).

### 3.2 Motif, Conserved Domain, and Cis‐Acting Element Analyses

To characterize the structural features of WOX proteins and assess their conservation and divergence among evolutionary clades, motif analysis was performed. The results showed that motif 1 and motif 2 were present in all alfalfa WOX proteins and together constituted the core functional domain of WOX proteins, namely the homeodomain (indicated by the gray bar in Figure S4).

Distinct motif architectures were observed among different clades. Proteins in the WUS clade contained only motifs 1 and 2. Members of the ancient clade harbored motifs 1, 2, and 10, with motif 10 consistently located at the N‐terminus. In contrast, proteins in the intermediate clade exhibited the most complex motif composition, typically containing multiple clade‐specific motifs, such as motifs 3 and 7, which were predominantly distributed in the C‐terminal region.

**FIGURE 3:**
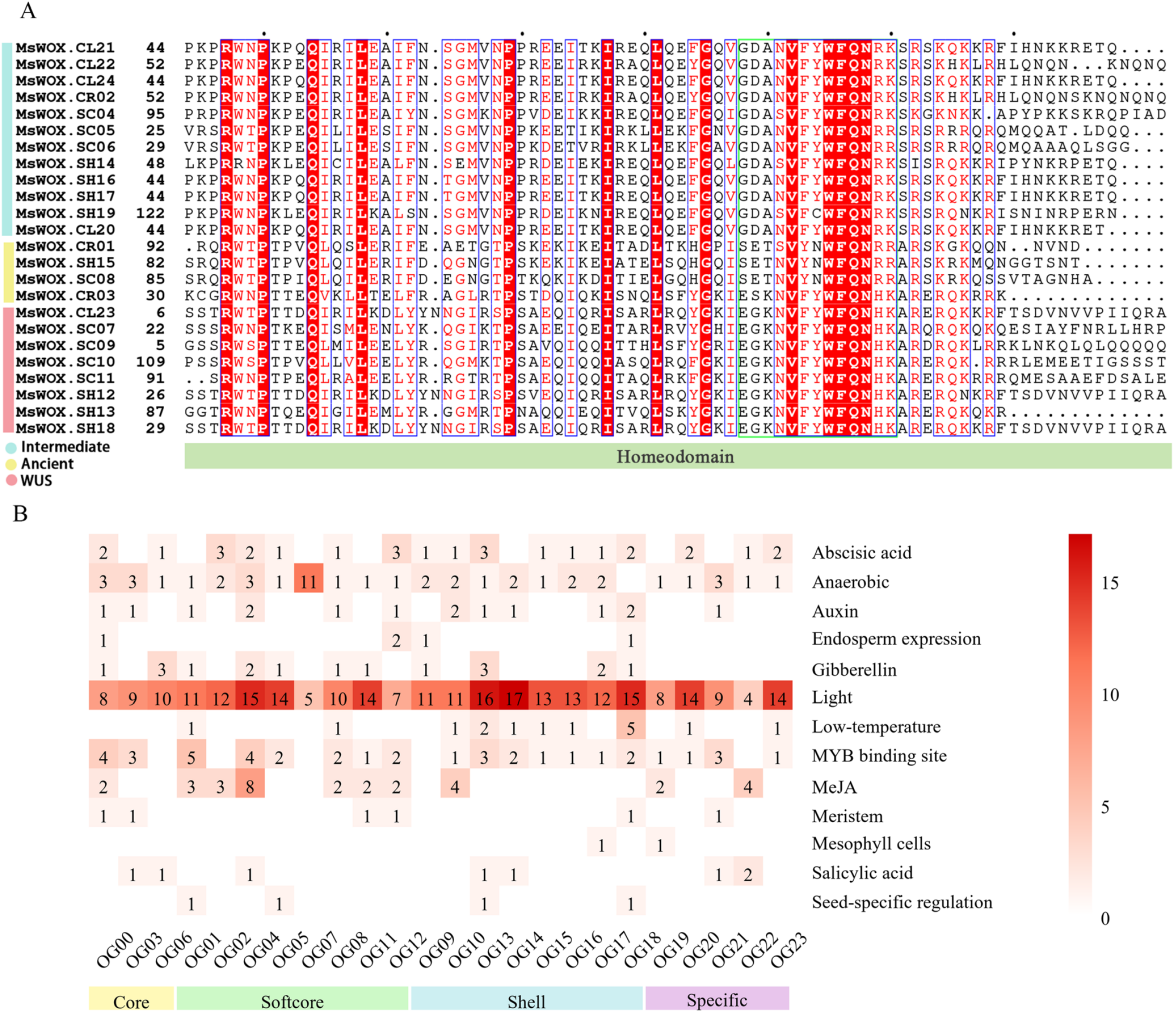
(A) Multiple sequence alignment of representative genes from different OGGs in alfalfa. (B) Average number of cis‐acting regulatory elements in representative genes from each OGG.

Multiple sequence alignment revealed a high degree of overall sequence conservation across the *WOX* gene family, particularly within the homeodomain, where representatives from different OGGs showed strong sequence consistency (Figure 3A). Further comparison indicated that sequence divergence among different subfamilies was mainly concentrated within amino acid positions 45–57 of the homeodomain. Within this region, each evolutionary clade displayed stable and clade‐specific signature residue combinations. The intermediate clade was characterized primarily by glycine, aspartic acid, and alanine residues, whereas the ancient clade showed a combination dominated by glutamic acid, variable residues, and lysine. In contrast, the WUS clade was mainly characterized by serine, glutamic acid, and threonine residues.

To further investigate the potential regulatory features, cis‐elements within the upstream promoter regions of each OGG were systematically analyzed (Figure 3B). Light‐responsive elements were the most abundant across all OGG types, indicating their widespread distribution within the *WOX* gene family. Core OGGs contained relatively fewer light‐responsive elements and lacked cis‐elements associated with mesophyll cell expression and low‐temperature responsiveness. In specific OGGs, cis‐elements related to endosperm expression, gibberellin responsiveness, and seed‐specific regulation were absent.

At the individual OGG level, methyl jasmonate (MeJA)‐responsive elements were most abundant in OG04. Anaerobic‐responsive elements were present in 23 of the 24 OGGs, with the exception of OG18, and OG07 exhibited the highest average number of anaerobic‐responsive elements, with a mean of 11.

### 3.3 Gene Duplication and Expansion Analysis

**FIGURE 4:**
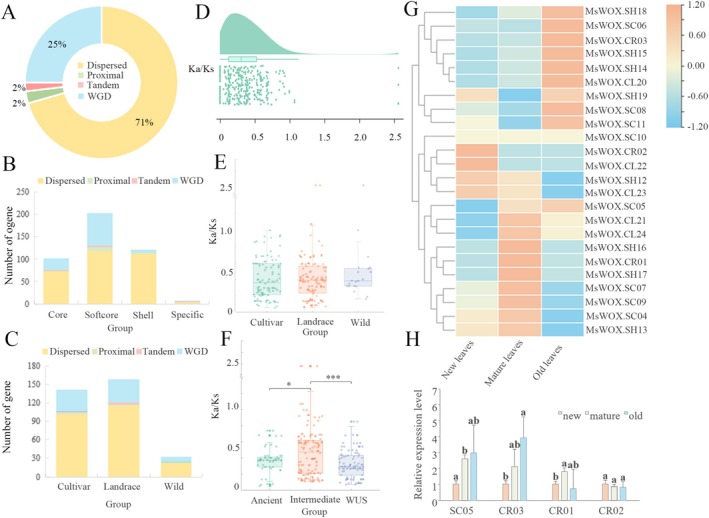
(A) Percentage of genes in each copy type; (B) Number of genes of different copy types in each OGG; (C) Number of genes of different copy types within each type; (D) Ka/Ks distribution of alfalfa *WOX* gene family members; (E) Ka/Ks variation of *WOX* genes across different domestication types; (F) Ka/Ks variation of *WOX* genes in each clade (*p < 0.05; ***p < 0.001); (G) Expression levels of representative OGG genes during leaf development; (H) Expression patterns of four representative genes in leaves at different developmental stages, including young, mature, and old leaves.

To elucidate the expansion mechanisms of the *WOX* gene family in alfalfa, duplication types were classified based on 2623 homologous gene pair relationships (Figure 4A). No singleton genes were detected. Dispersed duplication was the most prevalent, accounting for 305 genes and approximately 71% of all *WOX* genes. Whole‐genome duplication (WGD) was the second‐most frequent duplication type, accounting for 108 genes (approximately 25%). In contrast, tandem and proximal duplications were relatively rare, each contributing approximately 2% of the total genes (Figure 4A).

Distinct duplication patterns were observed among different OGG categories within the WOX pangenome. In core OGGs, 73 genes originated from dispersed duplication and 26 genes from WGD. Soft‐core OGGs exhibited the greatest expansion, driven primarily by dispersed duplication (117 genes) and WGD (74 genes). In shell OGGs, dispersed duplication remained the dominant mode, contributing 112 genes. Specific OGGs contained the fewest genes, which were generated through three dispersed duplications, two tandem duplications, and one WGD event (Figure 4B).

Duplication patterns were further compared among cultivated varieties, landraces, and wild accessions. Overall, highly consistent duplication‐type distributions were observed across all domestication groups. Dispersed duplication was the most abundant type in both cultivated varieties and landraces, with 101 genes in cultivated varieties and 115 in landraces. The number of WGD‐derived genes was comparable between cultivated varieties (37 genes, 36.6%) and landraces (38 genes, 33%). In wild accessions, *WOX* gene expansion was mainly driven by dispersed duplication and WGD, with only a single gene derived from proximal duplication (Figure 4C).

### 3.4 Selection Pressure Analysis of the *WOX* Gene Family

Selection pressure acting on *WOX* genes was assessed by calculating Ka/Ks ratios for all 432 genes using their closest homologs (Table S6). The majority of *WOX* gene pairs exhibited Ka/Ks values close to zero, indicating strong purifying selection across the gene family. Only two gene pairs displayed Ka/Ks values greater than 2. Notably, the gene *B62g83255.1.p1* from OGG OG0000001 showed the highest Ka/Ks value (2.56), suggesting that there may be relatively strong selection pressures (Figure 4D). Homology analysis indicated that both *B62g83255.1.p1* and *B62g81239.p1* were putative homologs of *AtWOX9*. Further alignment of the conserved homeodomain revealed that *B62g81239.p1* was completely identical to *MtWOX9*, while *B62g83255.1.p1* carried seven amino acid substitutions in this region (Figure S5).

Clear differences in selection pressure acting on the *WOX* gene family were observed among different domestication types (Figure 4E). Cultivated varieties exhibited the lowest mean Ka/Ks value (0.40), followed by landraces (0.43), while the wild accessions showed the highest value (0.53). Overall, average Ka/Ks values increased with decreasing levels of domestication, and genes with relatively high Ka/Ks values were predominantly distributed in landraces and wild accessions.

Selection pressure was further compared among evolutionary clades (Figure 4F). The ancient clade had a mean Ka/Ks of 0.37, whereas the WUS clade had the lowest mean (0.32). In contrast, the intermediate clade exhibited the highest mean Ka/Ks value (0.50), indicating stronger selective pressure. Consistent with this pattern, gene pairs with Ka/Ks values > 2 were exclusively clustered within the intermediate clade.

### 3.5 Pangenome‐Based Transcriptomic Analysis

Leaf senescence is a critical developmental process in alfalfa that directly affects photosynthetic duration, biomass accumulation, and forage quality during late growth stages. Dissecting the transcriptional regulation of senescence‐associated genes is therefore essential for improving yield stability and nutritional value in perennial forage crops.

To investigate dynamic changes in *WOX* gene expression during alfalfa leaf senescence, 43 leaf transcriptome datasets representing young, mature, and senescent leaves were analyzed (Figure 4G; Table S7). Following row‐wise scaling of the pangenome‐based expression matrix, representative genes from different OGGs exhibited distinct expression trends across the three developmental stages.

Overall, most OGG representatives showed increased expression in mature and senescent leaves, whereas only *CR02* and *CL22* displayed pronounced upregulation in young leaves. Notably, the three core representatives exhibited clear stage‐specific patterns: *CR01* was preferentially expressed in mature leaves, *CR02* showed higher expression in young leaves, and *CR03* was upregulated in senescent leaves. In addition, soft‐core representatives were predominantly upregulated in mature and senescent leaves, while relatively few representatives showed elevated expression in young leaves.

We selected three core genes, *CR01*, *CR02*, and *CR03*, together with SC05, a gene showing significant expression variation in the pan‐transcriptome, based on OGG classification, cross‐species homology analysis, and pan‐transcriptomic expression patterns. These genes were then subjected to qRT‐PCR validation. The results showed that the relative expression patterns of *SC05*, *CR01*, and *CR03* were generally consistent with those observed in the pan‐transcriptome, and each of them exhibited relatively high expression at a specific stage of leaf development (Figure 4H). *CR02* also showed an expression pattern consistent with the pan‐transcriptomic data, although the magnitude of change was relatively small. Overall, the qRT‐PCR results validated the reliability of the pan‐transcriptomic expression analysis. Given that leaf developmental status and senescence progression are closely associated with photosynthetic duration, biomass accumulation, and forage quality, these conserved WOX members may function as stable regulatory factors in fundamental leaf developmental processes.

## 4 Discussion

### 4.1 Evolutionary Stability and Population‐Level Divergence of the *WOX* Gene Family in Alfalfa

Based on the recently released alfalfa pangenome dataset, this study provides a systematic characterization of the composition, evolutionary divergence, and expansion patterns of the *WOX* gene family at both the OGG and population levels. Our analyses demonstrate that the alfalfa *WOX* gene family is organized around a conserved genetic framework, complemented by a smaller set of variable genes, and displays differential evolutionary stability and plasticity across evolutionary clades and domestication types.

Only minor differences in the number of *MsWOX* genes were observed among accessions with different material types and geographical origins. Moreover, PCA based on WOX OGG gene counts showed no obvious separation among germplasm types, further indicating that the *WOX* gene family is highly conserved in alfalfa. Core and soft‐core *WOX* genes together account for more than 70% of the total family size, indicating that the majority of *WOX* genes have been stably retained during alfalfa evolution. WOX transcription factors play central roles in fundamental developmental processes such as meristem maintenance and developmental phase transitions, which are typically subject to strong evolutionary constraints on protein structure and function (Du et al. 2025). Accordingly, the predominance of core and soft‐core WOX members suggests that these genes constitute the structural and functional backbone of the *WOX* gene family in alfalfa, providing a stable genetic foundation for normal plant growth and development.

At the clade level, distinct evolutionary patterns were observed. The WUS clade contained a higher proportion of soft‐core and shell genes compared with the ancient clade, suggesting greater genetic plasticity among WUS‐type *WOX* genes. Previous studies have reported that WUS‐clade genes are frequently involved in highly dynamic developmental processes, such as regeneration, and their expression and functional states are often sensitive to environmental conditions and domestication backgrounds (Cao et al. 2015). Such regulatory flexibility may facilitate increased variability in gene composition across accessions, consistent with the evolutionary patterns observed in this study. Moreover, phylogenetic analysis revealed that the intermediate and WUS clades contain substantially more WOX members in alfalfa than in *Arabidopsis thaliana* , suggesting clade‐specific expansion (Bhattacharjee et al. 2015; Lian et al. 2014). Considering the perennial growth habit of alfalfa, such expansion may be associated with enhanced regenerative capacity and environmental adaptability required over repeated growth life cycles (Kadri et al. 2021).

At the population level, comparative pangenome analysis among cultivated varieties, landraces, and wild alfalfa accessions revealed only minor differences in the numbers of core, soft‐core, and shell genes, indicating that the overall composition of the *WOX* gene family has remained relatively stable across domestication types. Although wild species are often reported to harbor more specific genes due to higher genetic diversity (Liu et al. 2019), specific *WOX* genes in this study were predominantly detected in landraces rather than in wild accessions. This pattern may partly reflect sampling effects but may also indicate adaptive differentiation of landraces during long‐term regional cultivation in response to local environmental conditions and agronomic requirements (Lippmann et al. 2019).

Structurally, the WOX homeodomain was highly conserved across all subfamilies. This domain spans approximately 60 amino acids and consists of three α‐helices, with the third α‐helix playing a pivotal role in sequence‐specific DNA recognition (Li et al. 2024). Despite overall conservation, several amino acid positions, such as residue 45, exhibited clade‐specific variation, which may contribute to the subtle functional diversification among WOX proteins. However, the biological significance of these variations remains to be elucidated through further functional studies.

Consistently, motif 1 and motif 2 were conserved across all three WOX clades. Motif 1 corresponds largely to the helix 2‐helix 3 region of the homeodomain responsible for DNA binding, whereas motif 2 encompasses the N‐terminal region, including helix 1 and its linker. Together, these motifs ensure structural stability and transcriptional regulatory activity of WOX proteins (Gehring et al. 1994). Collectively, these findings indicate that the homeodomain is under strong purifying selection and represents an essential structural unit in the evolutionary history of the *WOX* gene family.

Overall, the alfalfa *WOX* gene family has maintained a highly conserved core while simultaneously accumulating genetic diversity through clade‐specific expansion and population‐level differentiation. This balance between evolutionary conservation and diversification provides a molecular basis for the flexible regulation of WOX‐mediated developmental programs across diverse ecological and developmental contexts in this perennial forage crop.

### 4.2 Duplication Patterns of the *WOX* Gene Family in Alfalfa

The expansion mechanisms of the *WOX* gene family exhibit considerable diversity. For example, *WOX* genes in rose have predominantly expanded through whole‐genome and segmental duplication events (Duan et al. 2024), whereas segmental duplication is the dominant driver in loquat (Yu et al. 2022). These differences highlight that WOX family expansion is not conserved by species‐specific evolutionary histories, genome architecture, and life‐history traits.

In alfalfa, no singleton *WOX* genes were detected, and most family members originated from dispersed duplication and WGD. This pattern suggests that these two duplication modes represent the principal drivers of WOX family expansion in alfalfa. At the OGG levels, WGD‐derived genes mainly contributed to the core and soft‐core categories, whereas their contribution to the shell category was markedly lower. This observation suggests that genes generated through large‐scale duplication events are more likely to be retained over long evolutionary timescales, forming stable sets (Panchy et al. 2016). In contrast, dispersed duplication appears to generate genes that are more prone to presence‐absence variation among accessions, resulting in genetic flexibility.

Importantly, duplication patterns were highly consistent across cultivated varieties, landraces, and wild accessions, including that the fundamental expansion mechanisms of the *WOX* gene family have remained largely unchanged during alfalfa domestication and improvement. This evolutionary stability further supports the notion that *WOX* genes represent a conserved developmental module in alfalfa (Cao et al. 2025).

### 4.3 Selection Pressure and Agronomic Implications of *WOX* Gene Divergence in Alfalfa

Ka/Ks analysis revealed that most *WOX* gene pairs exhibit ratios below 1, indicating that the *WOX* gene family has been subject to strong purifying selection. This pattern is consistent with essential developmental roles of WOX transcription factors, whose CDS are expected to be highly conserved. At the population level, higher average Ka/Ks values in landraces and wild accessions may reflect exposure to more diverse and heterogeneous selective environments. In contrast, cultivated varieties have undergone prolonged artificial selection, during which key developmental genes tend to be conserved to maintain agronomic stability (Meyer and Purugganan 2013; Murphy 2007). Similar trends have been reported in pangenome‐based analyses of other crops, such as barley (Bai et al. 2025), supporting the generality of this pattern.

Notably, B62g83255.1.p1 exhibited the highest Ka/Ks value among the *WOX* genes identified in this study, suggesting that this gene may have undergone relatively rapid sequence divergence. Phylogenetic analysis indicated that B62g83255.1.p1 is homologous to *WOX9* members. Previous studies in the model legume *Medicago truncatula* showed that *MtWOX9* antagonistically regulates STF/LAM1‐mediated leaf blade expansion and participates in balancing cell proliferation and differentiation through CKX3‐mediated cytokinin homeostasis (Wang et al. 2022). Considering that leaf size, leaf blade expansion, and leaf‐to‐stem ratio are important agronomic traits affecting photosynthetic efficiency, biomass accumulation, and forage quality in alfalfa, the rapid divergence of B62g83255.1.p1 may reflect its potential involvement in leaf morphogenesis, plant architecture optimization, or developmental differentiation. Moreover, the WOX9‐related regulatory module may be sensitive to expression dosage and regulatory context, which could impose distinct selective pressures on *WOX9* homologs in different genetic backgrounds. Therefore, B62g83255.1.p1 may represent an important candidate gene for further functional studies of WOX9‐mediated regulation of leaf development and yield‐related traits in alfalfa.

### 4.4 Expression Dynamics of the *WOX* Gene Family in the Alfalfa Pangenome

By integrating transcriptomic data from young, mature, and senescent leaves, this study systematically characterized the expression dynamics of *MsWOX* genes during leaf development and senescence. The results showed that different *MsWOX* members exhibited clear stage‐biased expression patterns across different leaf developmental stages. Further qRT‐PCR validation showed that the expression trends of *CR01*, *CR02*, *CR03*, and *SC05* in young, mature, and senescent leaves were generally consistent with the pan‐transcriptome results, further supporting expression‐level regulatory divergence of *MsWOX* genes during leaf development (Li et al. 2022).

Notably, the three core *WOX* genes exhibited complementary expression patterns, with preferential expression in young, mature, and senescent leaves, respectively (Figure 4G). These results suggest that core MsWOX members exhibit stage‐biased expression patterns during leaf development and senescence, which may be related to photosynthetic duration, biomass accumulation, and forage yield (Xu et al. 2023). The qRT‐PCR results showed that *CR02* exhibited relatively moderate expression changes. This relatively stable expression pattern may indicate that *CR02* is not a strongly stage‐inducible WOX member during leaf development and senescence, but may instead function as a conserved component involved in basal developmental regulation.

*MsWOX.SH13* was highly expressed in young and mature leaves but was markedly downregulated in senescent leaves. Phylogenetic analysis and multiple sequence alignment showed that *MsWOX.SH13* is homologous to Arabidopsis *AtWOX4* and rice *OsWOX4*. This expression pattern is consistent with the reported functions of *AtWOX4* and *OsWOX4*, which are involved in maintaining meristematic activity, promoting cell proliferation, and preventing premature cell differentiation (Figure S6) (Yasui et al. 2018). Similarly, *MsWOX.SC07*, corresponding to *AtWOX2*, exhibited reduced expression in senescent leaves, consistent with the role of *AtWOX2* in early developmental patterning (Breuninger et al. 2008).

In addition, *MsWOX.SH16*, *MsWOX.SH17*, and *MsWOX.SC04* (corresponding to *WOX8*) displayed high expression in young leaves, moderate expression in mature leaves, and a rapid downregulation during senescence, suggesting potential involvement in early developmental processes. In contrast, *MsWOX.SC09* (corresponding to *WOX3*) showed elevated expression in mature leaves, consistent with the role of *AtWOX4* in lateral organ development (Shimizu et al. 2008). Finally, genes such as *MsWOX.SC05*, which were upregulated during senescence, represent promising candidates for future functional studies aimed at elucidating the molecular mechanisms by which WOX transcription factors regulate leaf aging in alfalfa.

## 5 Conclusion

In this study, we systematically characterized the *WOX* gene family in alfalfa within a pangenome framework, providing comprehensive insights into gene composition, evolutionary divergence, and expansion mechanisms. Our analyses show that the *WOX* gene family is dominated by core and soft‐core members, reflecting a high degree of evolutionary stability and forming a fundamental regulatory framework that supports essential developmental processes in alfalfa. Distinct evolutionary clades displayed pronounced differences in gene composition, duplication patterns, and selective pressures. Dispersed duplication emerged as the primary driver of WOX family expansion, whereas whole‐genome duplication preferentially contributed to the long‐term retention of conserved members. Ka/Ks analysis indicated that most *WOX* genes were subject to strong purifying selection, suggesting overall evolutionary conservation of the *WOX* family in alfalfa. However, several genes, particularly *MsWOX9*, exhibited relatively elevated selective pressure, highlighting their potential roles in domestication‐related divergence and agronomically relevant trait variation. Pan‐transcriptome analysis, supported by qRT‐PCR validation, further revealed that representative *MsWOX* genes exhibited stage‐biased expression patterns in young, mature, and senescent leaves, suggesting expression‐level differences during leaf development and senescence. These results further indicate that different *MsWOX* members may have potential regulatory effects at distinct stages of leaf growth.

Collectively, this study advances our understanding of the evolutionary dynamics of the *WOX* gene family and provides a valuable genomic foundation for future studies and molecular breeding of this important perennial forage crop.

## Author Contributions

**Hao Wen:** formal analysis (equal), methodology (equal), software (equal), writing – original draft (equal). **Yiwei Bai:** formal analysis (equal), methodology (equal), software (equal), writing – review and editing (equal). **Zhao Guo:** investigation (equal), software (equal). **Wenwu Qian:** software (equal). **Yizhi Huang:** validation (equal). **Yutao Liang:** visualization (equal). **Guodong Yang:** data curation (equal). **Yunwei Zhang:** funding acquisition (equal), project administration (equal), resources (equal).

## Funding

The project title is the Major Demonstration Project of “Open Competition” for Seed Industry Science and Technology Innovation in Inner Mongolia (Grant No. 2022JBGS0016).

## Conflicts of Interest

The authors declare no conflicts of interest.

## Supporting information


**Figure S1:** Distribution of MsWOX gene numbers among alfalfa accessions with different improvement statuses and geographic origins. (A) Average number of MsWOX genes in accessions with different improvement statuses. (B) Average number of MsWOX genes in accessions from different geographic regions.
**Figure S2:** Variation in WOX OGG composition among alfalfa accessions with different improvement statuses.
**Figure S3:** Circular phylogenetic tree of WOX family proteins from alfalfa and representative plant species.
**Figure S4:** Motif composition and conserved domain architecture of representative WOX genes in *Arabidopsis thaliana* and alfalfa ( *Medicago sativa* ). The left panel shows the distribution of conserved motifs, and the right panel shows the distribution of the homeodomain.
**Figure S5:** Multiple sequence alignment of MsWOX9 candidate proteins and WOX9 homologs from *Medicago truncatula* and *Arabidopsis thaliana* .
**Figure S6:** Multiple sequence alignment of MsWOX.SH13 and WOX4 homologs from *Arabidopsis thaliana* and *Oryza sativa* .

**Table S1:** Detailed information of 24 alfalfa germplasm accessions.
**Table S2:** SRR and SRS accession numbers of *Medicago sativa* sequencing data in the NCBI SRA database.
**Table S3:** Primers used in this study.
**Table S4:** Orthogroup classification, pan‐genome category, and phylogenetic clade information of the WOX gene family in 24 alfalfa accessions.
**Table S5:** Detailed information of representative genes for each orthogroup (OGG).
**Table S6:** Ka/Ks analysis of WOX genes in alfalfa.
**Table S7:** Pan‐transcriptome expression profiles across different leaf samples.

## Data Availability

All data generated or analyzed during this study are included in this published article and its Supporting Information files.
