# Supplementary material for: Pangenome and Pan‐Transcriptome Analysis of the WOX Gene Family Reveals Evolutionary Conservation and Diversification in Alfalfa
Source: Ecol Evol. 2026 Aug 4;16(8):e74048. doi: 10.1002/ece3.74048 (PMC13435375; doi:10.1002/ece3.74048)
Supplement: Supplementary file 1 [file ECE3-16-e74048-s002.pdf]

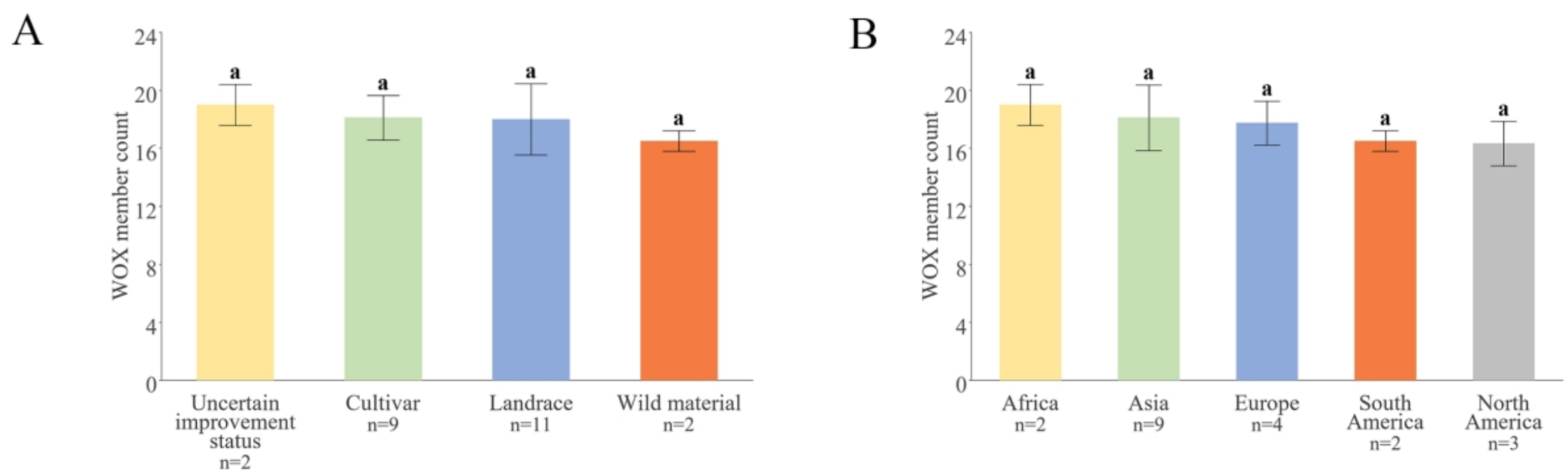

Supplement Fig 1. Distribution of MsWOX gene numbers among alfalfa accessions with different improvement statuses and geographic origins. (A): Average number of MsWOX genes in accessions with different improvement statuses. (B): Average number of MsWOX genes in accessions from different geographic regions.

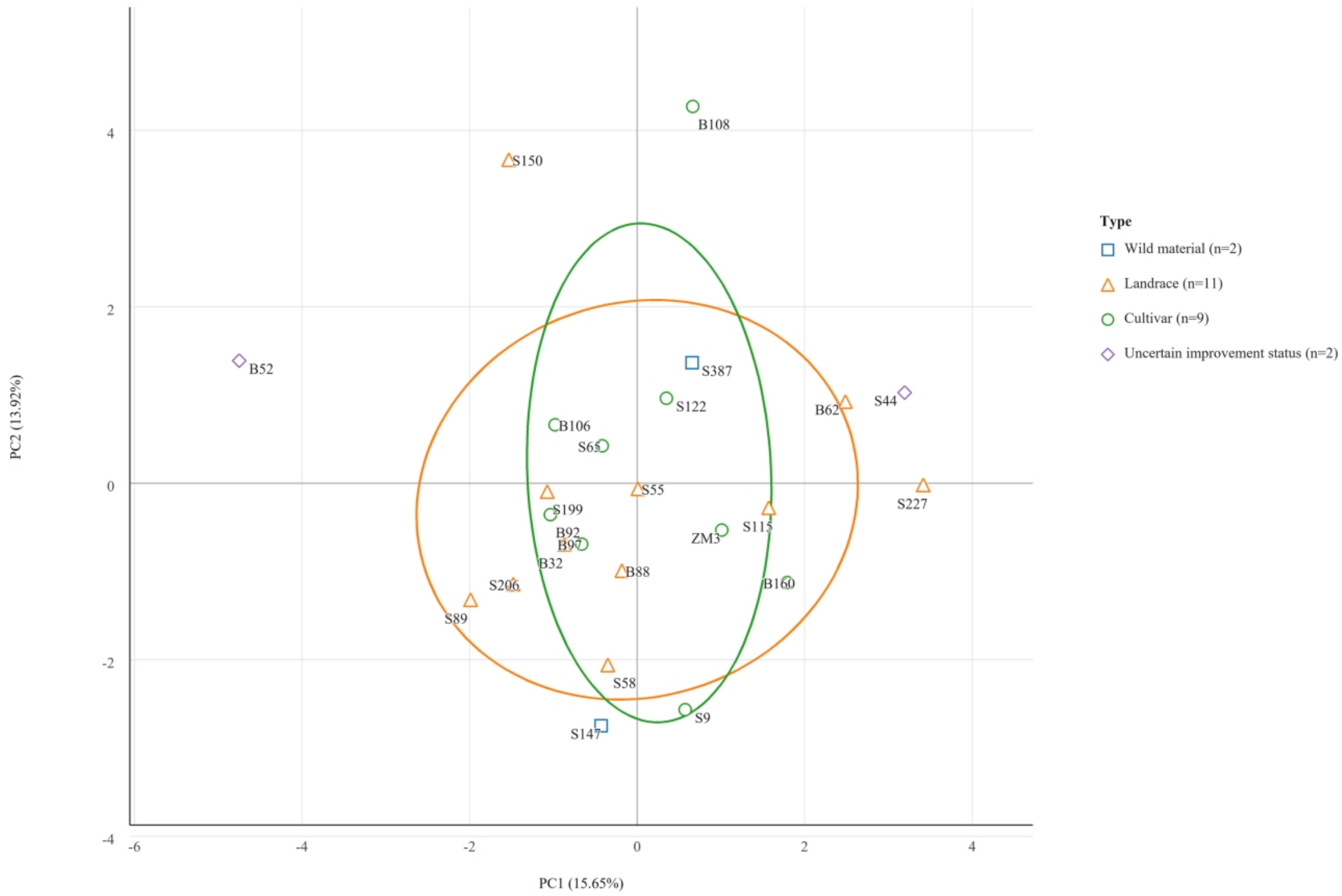

Supplement Fig 2. Variation in WOX OGG composition among alfalfa accessions with different improvement statuses.

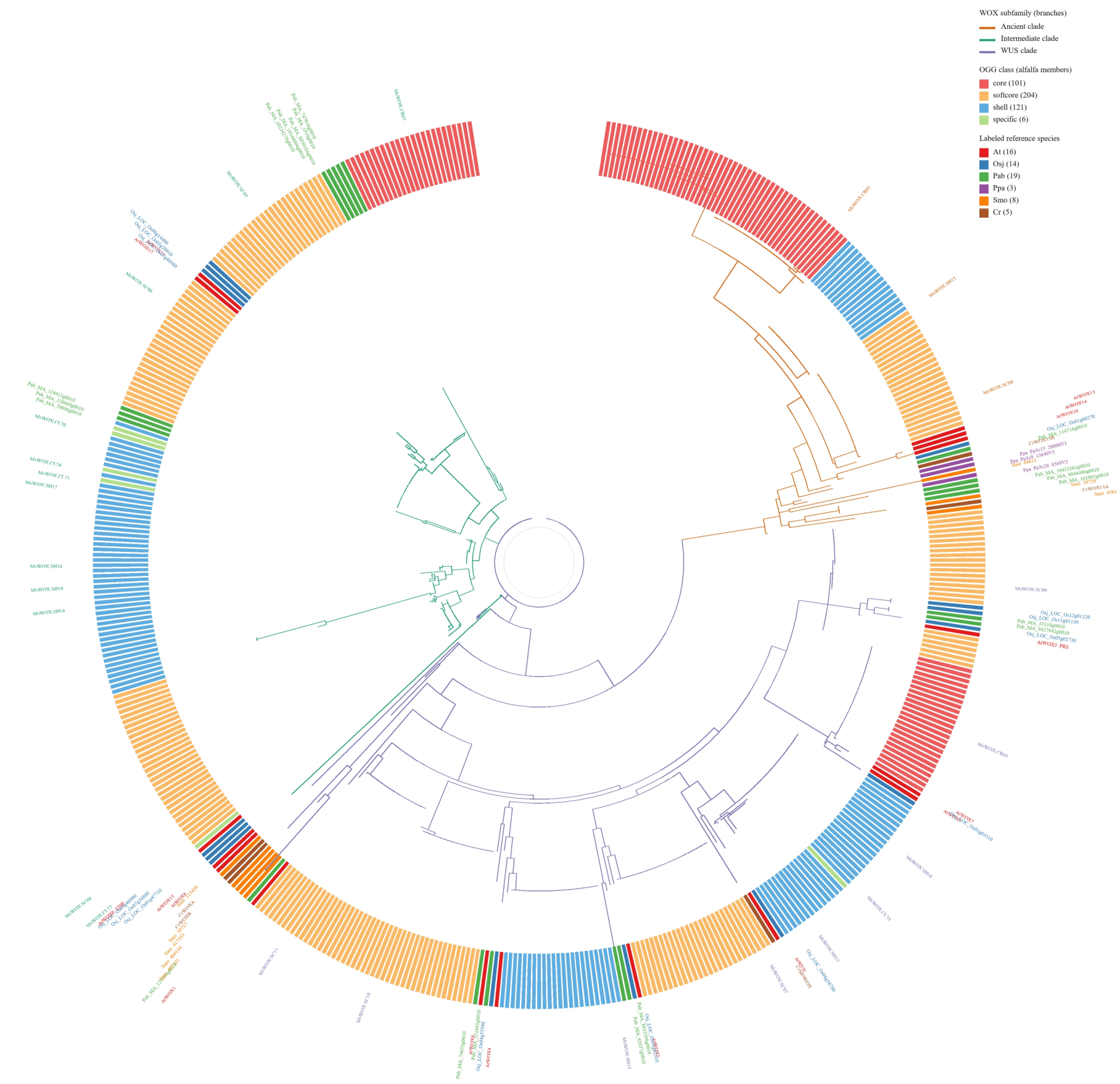

Supplement Fig 3. Circular phylogenetic tree of WOX family proteins from alfalfa and representative plant species.

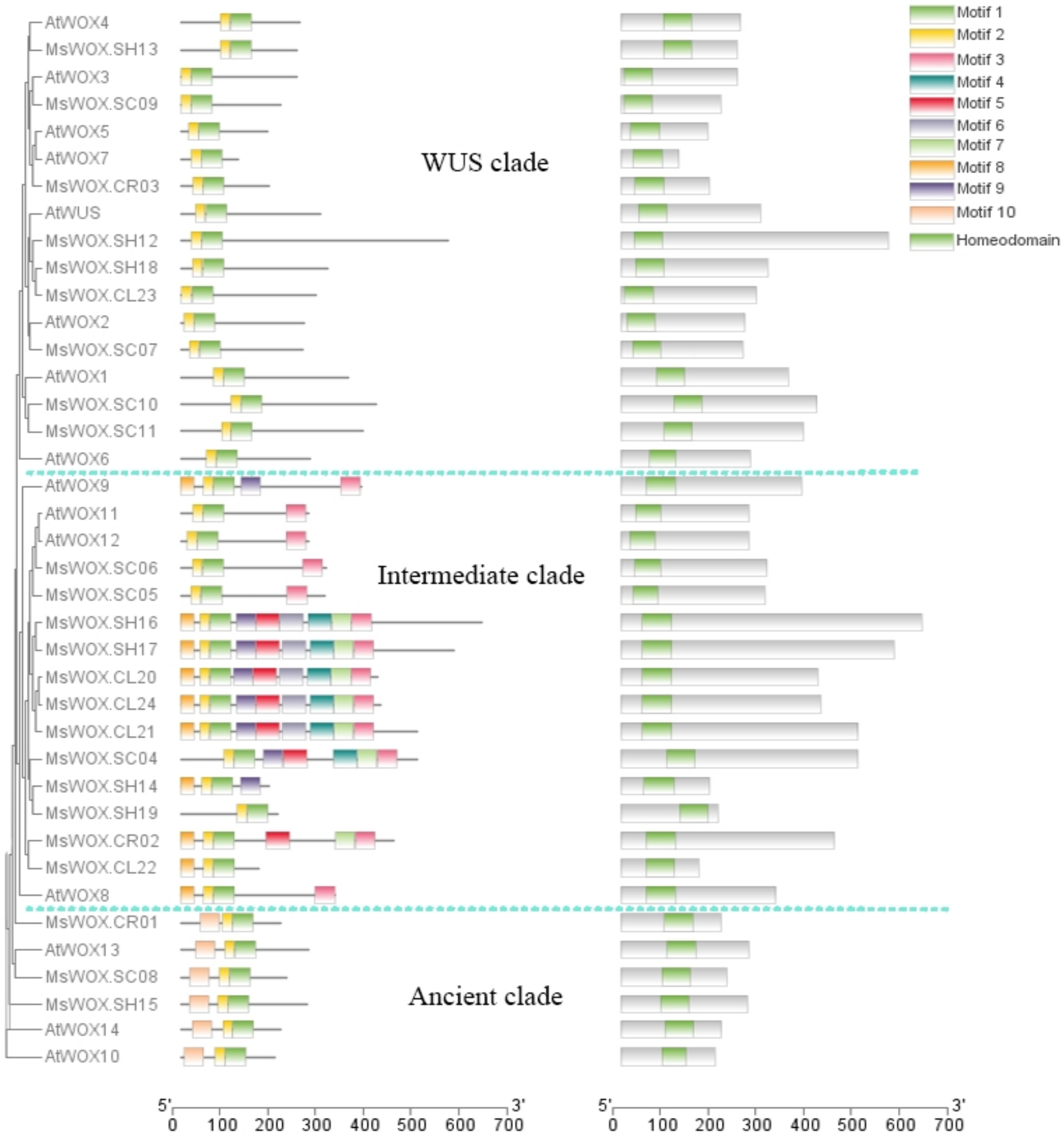

Supplement Fig 4. Motif composition and conserved domain architecture of representative *WOX* genes in *Arabidopsis thaliana* and alfalfa (*Medicago sativa*). The left panel shows the distribution of conserved motifs, and the right panel shows the distribution of the homeodomain.

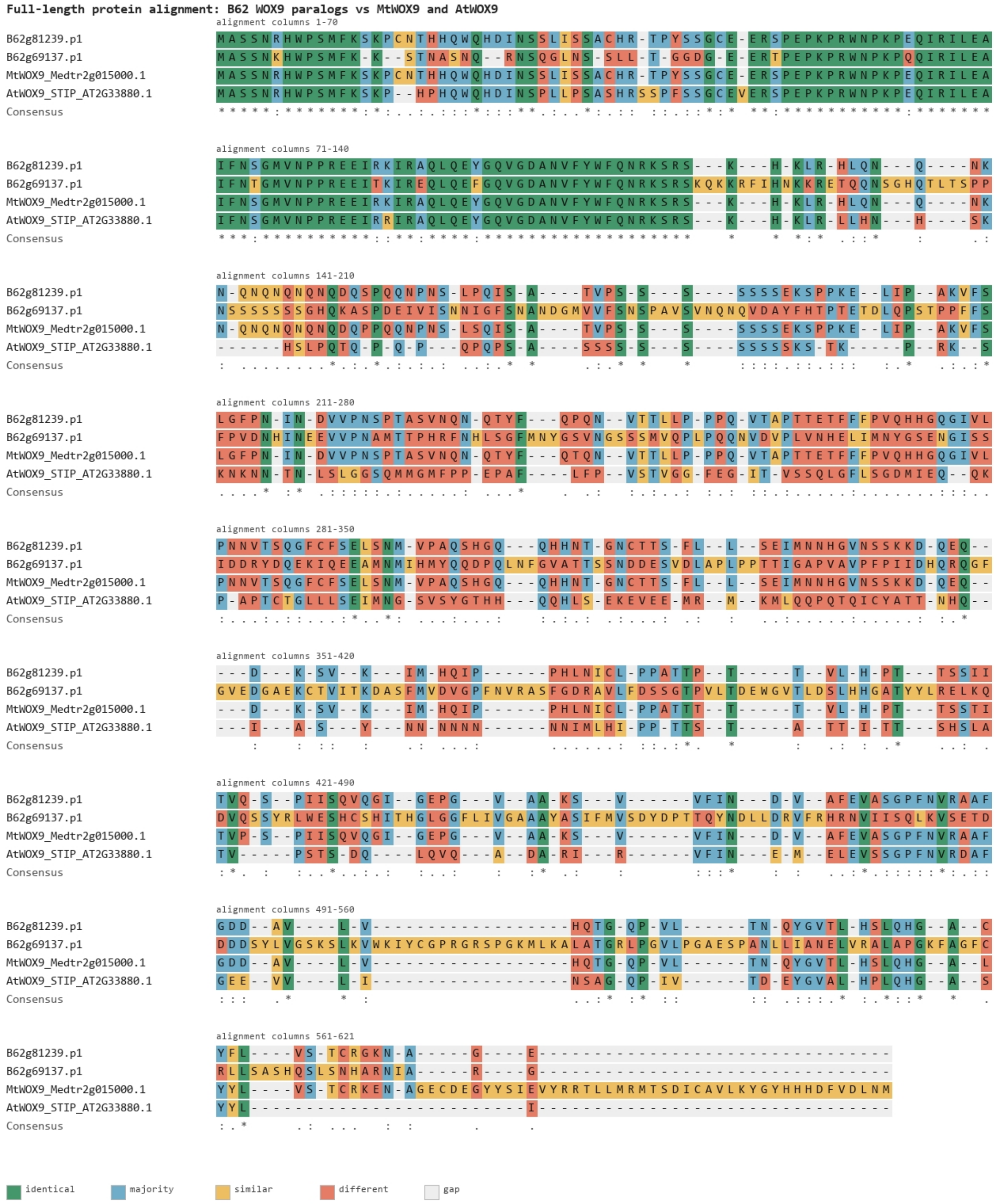

Supplement Fig 5. Multiple sequence alignment of MsWOX9 candidate proteins and WOX9 homologs from Medicago truncatula and Arabidopsis thaliana.

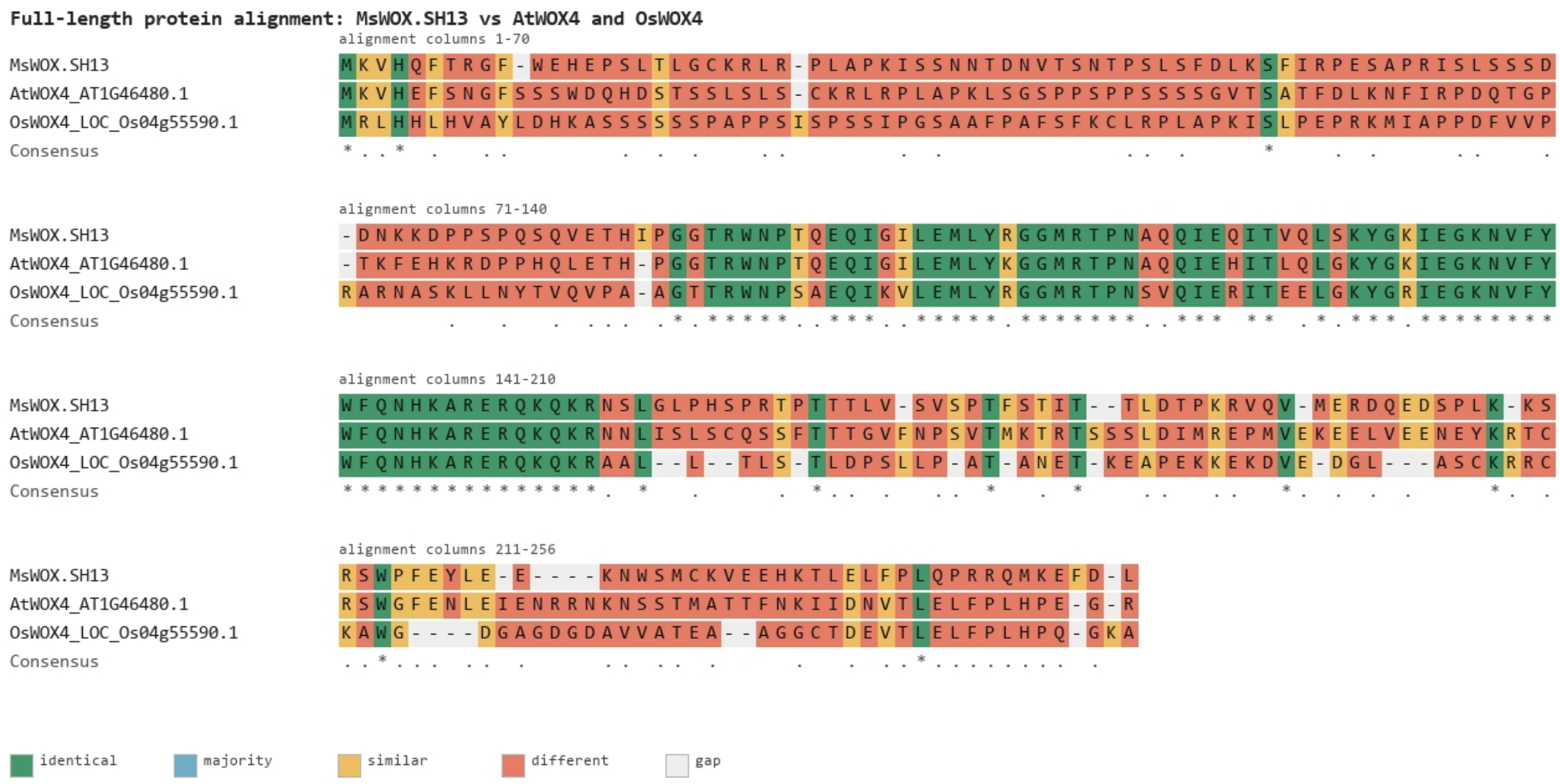

Supplement Fig 6. Multiple sequence alignment of MsWOX.SH13 and WOX4 homologs from Arabidopsis thaliana and Oryza sativa.
